# Ampoule Synthesis of Na-Doped Complex Bromide Cs_2_AgBiBr_6_ with Double Perovskite Structure

**DOI:** 10.3390/ma18061197

**Published:** 2025-03-07

**Authors:** Nigina K. Nosirova, Rustam K. Kamilov, Maqsudjon M. Ibrohimov, Leonid S. Lepnev, Mikhail O. Astafurov, Alexander V. Knotko, Anastasia V. Grigorieva

**Affiliations:** 1 Department of Material Science, Lomonosov Moscow State University, 119991 Moscow, Russia; 2 P.N. Lebedev Physical Institute of the Russian Academy of Sciences, 119991 Moscow, Russia; 3 Department of Chemistry, Lomonosov Moscow State University, 119991 Moscow, Russia

**Keywords:** double perovskite, Cs_2_NaBiBr_6_, Cs_2_AgBiBr_6_, solid-phase synthesis, melt crystallization, diffuse reflectance spectroscopy, photoluminescence spectroscopy

## Abstract

Compounds of the general composition A_2_B^I^B^III^X_6_ with a double perovskite (elpasolite) structure are currently considered as an alternative to lead halide perovskites APbX_3_ in electronics and photovoltaics due to their greater compositional flexibility and low toxicity. One such alternative is the recently synthesized double perovskite Cs_2_AgNaBiBr_6_ and a number of various substituted compounds. The close values of the radii of silver and sodium cations make tuning the optoelectronic properties of the double perovskite via the substitution of Ag^+^ by Na^+^ promising if the formation of the substitution solid solution Cs_2_Ag_1−x_Na_x_BiBr_6_ takes place. We explored different possible routes for the synthesis of this class of materials, including solid-phase or melt crystallization ampoule syntheses. Varying heating temperature and duration and using standard cooling processing or a quenching-like process, we demonstrate the instability of Cs_2_NaBiBr_6_ and Na-substituted compounds Cs_2−x_Na_x_AgBiBr_6_ in the temperature range of 300–650 °C. It is worth noting that the formation of Cs_2_Ag_1−x_Na_x_BiBr_6_ solid solutions by a solid-phase method is more favorable.

## 1. Introduction

In recent decades, perovskites have attracted much attention from researchers in the fields of material science and solid-state physics. These structures can be described by the formula ABX_3_, which represents a class of compounds where the cations A and B occupy cuboctahedral and octahedral positions, respectively, while anions represented by X surround them [1,2]. Double perovskite halides have unique structural and physical properties that make them attractive for various applications. For example, such materials are in demand within solar cells as light absorbers and electronic conductors [3,4]; they can be applied in catalysis and photocatalysis [5] or in other ways [6]. Due to their unique properties, double perovskites possess great potential for the development of new technologies and the improvement of existing ones. The investigation of double perovskites has a long story and continues today, and it is still in the mainstream. At the same time, the synthesis of double perovskites is a complex process that requires certain conditions and control [7,8,9].

There are several methods for the synthesis of double halide perovskites, including solid-state synthesis, hydrothermal synthesis, melt crystallization, and even synthesis using radio-frequency heating [10,11,12]. Each method has its own advantages and limitations, and the choice of synthesis method depends mostly upon which requirements should be met by complex halide compounds.

The most investigated double perovskite halide with the composition Cs_2_AgBiBr_6_ is successfully obtained in the form of films [13], single crystals [14], and nano-sized products [7]. The structure includes silver and bismuth cations in the octahedral positions of [AgBr_6_] and [BiBr_6_], respectively. It has a bandgap of about 2.1 eV that works for optoelectronic applications [15]. It has been demonstrated also that Cs_2_AgBiX_6_ (X = Cl, Br) films reach power conversion efficiencies (PCEs) up to 2.5% and open circuit voltage values (*V_OC_*) above 1.0 V [16]. It has also been found that an uneven distribution of Ag^+^ and Bi^3+^ cations in octahedrons of bromine leads to a decrease in efficient bandgap values [15]. Conflicting results about the thermal stability of the Cs_2_AgBiBr_6_ phase were reported in Refs. [16,17], with some claiming stability from 300 to 400 °C based on X-ray diffraction and thermogravimetry studies and others up to 250 °C from Raman studies for Cs_2_AgBiBr_6_. 

At the same time, there is a promising double perovskite Cs_2_NaBiBr_6_ with a simulated bandgap of 2.09 eV [18]. The closeness of the values of ionic radii of Ag^+^ and Na^+^ enables the emergence of attractive substituted solid solutions with the composition Cs_2_Ag_1−x_Na_x_BiBr_6_ (x = 0–0.3) and further investigation of their optical properties for optoelectronic and other applications. Recently, Pistor et al. showed the possibility of substitution in a long solid-phase fusing at 250 °C [19]. Here, for the first time, we discuss the products of melt crystallization including quenching-like processing.

In this manuscript, we discuss the possibility of melt ampoule synthesis of double perovskite halides with the composition of Cs_2_AgBiBr_6_ and Cs_2_NaBiBr_6_. The possibility of the substitution of sodium at cationic positions A^+^ and/or B^+^ is considered, considering the conflicting information in the literature. Namely, Li et al. demonstrate the substitution of silver by sodium [20], while Wu et al. discuss the substitution of cesium by sodium in the double perovskite compound Cs_2_AgBiBr_6_ [21]. In addition to the phase analysis of the samples, their optical absorption spectra and photoluminescence spectroscopy are discussed below.

## 2. Experimental Section

### 2.1. Synthesis Technique

The synthesis of the double perovskite halide Cs_2_AgBiBr_6_ was carried out using CsBr (SigmaAldrich, Darmstadt, Germany, 99.99%), AgBr, and BiBr_3_ as precursors. The synthesis of hypothetic compound Cs_2_NaBiBr_6_ with a double perovskite structure was carried out using CsBr (Sigma, Darmstadt, Germany, 99.99%), NaBr («Reachim», Moscow, Russia, purum), and BiBr_3_ as precursors.

Bismuth tribromide was synthesized using a «wet chemistry» method. Namely, basic bismuth (III) carbonate (BiO)_2_CO_3_ (Chimkraft, Kaliningrad, Russia, >99%) was dissolved in HBr aqueous solution («Reachim», Moscow, Russia, p.a.) followed by water evaporation that finally produced yellow-colored polycrystalline bismuth tribromide which was washed with CCl_4_ using water jet pump with a glass filter and dried under vacuum conditions and under P_2_O_5._ According to XRD results, we obtained pure single-phase BiBr_3_ (Materialscloud, COD 8103605, Berkeley, CA, USA).

Synthesis of silver bromide was carried out by mixing AgNO_3_ («Reachim», Moscow, Russia, purum) and KBr («Ruschim», Moscow, Russia, purum) in an aqueous solution with the precipitation of a yellow crystalline product that was washed with distilled water and then dried in air conditions at 80 °C. According to XRD results, we obtained a single-phase product (PDF2 database file [79-149]).

For the synthesis of double perovskite compounds, we used 8 mm quartz ampoules filled with precursors layer by layer from bottom to top according to their volatilities (starting with the precursor having a lower boiling temperature). The ampoules were vacuumed and sealed under a pressure of 0.08 mmHg. Since bromides are highly volatile substances, to prevent their evaporation during the sealing, the ampoules were covered with asbestos clay and cooled with liquid nitrogen. Then, the ampoules were dipped into aluminum oxide powder (corundum Al_2_O_3_) and placed into a furnace for sintering.

Solid-phase syntheses (melt crystallization) were carried out by applying various fusing regimes. Solid-phase sintering and melt crystallization allow closed-volume synthesis to be performed. For this purpose, initial reagents were mixed in the form of powder utilizing stoichiometric quantities and ground in an agate mortar in an argon atmosphere in a glove box. The following temperature treatment mode was utilized: heating rate, 1°/min; T = 320–650 °C. A standard regime was used for the cooling of the furnace with the samples within it after the heater was switched off.

Sintering parameters, namely heating rate (1°/min and 5°/min), exposition temperature (320 °C, 500 °C, and 650 °C), and duration (20 h and 48 h), were varied to obtain the Cs_2_NaBiBr_6_ single-phase product. In the present work, we demonstrate our data for the heating rate of 1°/min and duration of fusing of 20 h because we observed no significant differences when these parameters were varied.

To obtain the Cs_2_Ag_1−x_Na_x_BiBr_6_ theoretical composition, samples were annealed at 300 °C for 24 h.

To obtain the Cs_2−2x_Na_x_AgBiBr_6_ composition, samples were annealed at 650 °C for a calcination time of 24 h. For this system, we analyzed the products of melt crystallization to reveal the role of the CsBr percentage in the melt and to compare the results with the wet-chemistry synthesis reported elsewhere [21].

Quenching-like processes were also utilized to obtain a sample with the theoretical composition of Cs_2_NaBiBr_6_. Ampoules were heated up to 650 °C and kept at this temperature for 2 h and then cooled down to 450 °C; afterward, the ampoules were quenched by water at room temperature (T = 24 °C) and opened right before their study using X-ray diffraction and optical spectroscopy methods.

### 2.2. Analysis Methods

X-ray diffraction (XRD) of the synthesized compounds was carried out using a Rigaku 2500 D-max diffractometer (Rigaku, Tokyo, Japan) with a point proportional detector and with a CuKα source (λ = 1.5418 Å) in the range 2θ = 5–60° with a step of 0.02°. Diffraction data were analyzed using specialized software, namely WinXPow (STOE & Cie, Version 1.07, Darmstadt, Germany) and Jana2006 (ECA-SIG#3, Prague, Chech Republic).

The microstructure and elemental composition of the samples were characterized by scanning electron microscopy (SEM) and energy-dispersive X-ray spectroscopy (EDS), respectively, using a Leo Supra 50 VP scanning electron microscope (LEO Carl Zeiss SMT Ltd., Oberkochen, Germany) with an energy-dispersive X-ray spectroscopy detector (Oxford Instruments, Oxford, UK) at a high accelerating voltage of 21 kV.

Photoluminescence (PL) spectra were recorded using an S2000 luminescence spectrometer (Ocean Optics, Orlando, FL, USA) with an LGI-21 pulsed nitrogen laser as an excitation source, using the following parameters: λ_ex_ = 337 nm, power ~2 mW, 100 pulses per second with 10 ns duration of each pulse. A KS-12 filter was used. Measurements were carried out at a temperature of 293 K and a wavelength range of 294–877 nm. The signal accumulation time was 6–6.5∙10^7^ ms.

Diffuse reflectance spectra (DRS) were obtained at 298 K using a Lambda 950 UV-visible spectrometer (PerkinElmer, Naperville, IL, USA). The spectral range was 400–800 nm with a scanning speed of 1 nm/s. Barium sulfate was applied as a standard.

## 3. Results and Discussion

### 3.1. Samples of General Composition Cs_2_NaBiBr_6_

**Figure 1:**
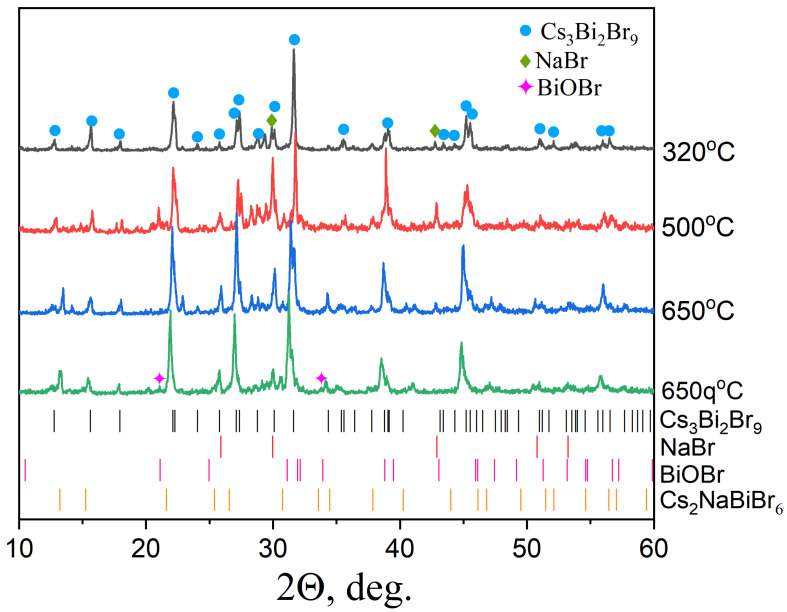
The XRD analysis of samples of the theoretical composition Cs_2_NaBiBr_6_ calcined at the temperatures 320–650 °C with standard cooling with a furnace, as well as at 650 °C with quenching in water from T = 450 °C (marked as 650q °C).

The samples of the theoretical composition Cs_2_NaBiBr_6_ obtained by the solid-phase and melt crystallization ampoule methods at temperatures of T = 320–650 °C were studied by X-ray diffraction to investigate their phase compositions (Figure 1). The XRD data demonstrate a formation of mixtures of two phases, namely bromobismuthate cesium Cs_3_Bi_2_Br_9_ (PDF-2 database file [70-493]) and NaBr (PDF-2 database file [72-1539]) in the entire sintering temperature range. The reflections of cesium bromobismuthate Cs_3_Bi_2_Br_9_ are sharp and originate from the high crystallinity of the binary bromide. The most intensive reflections are (100), (1-11) and (101), (002), (101) and (1-12), (003), (201) and (2-21), (2-22) and (202), (2-24) and (204). The most intensive reflections of sodium chloride observed in diffractograms are as follows: (111), (200), (220), (222). The reported-elsewhere BiOBr is found in low quantities (PDF-2 database file [9-393]). It exists in the samples as a product of hydrolysis of bismuth(III) compounds in air and is found in some products. No reflections of silicates are observed in the samples.

The presence of the sought-for Cs_2_NaBiBr_6_ phase in the sample is not confirmed. The primary crystallization of binary bromide Cs_3_Bi_2_Br_9_ or simple bromide NaBr in the reactionary melt probably prevents the growth of the Cs_2_NaBiBr_6_ phase by a significant decrease in the concentrations of cesium and bismuth or sodium. It is worth noting that the intensity of the NaBr reflections decreases with an increase in the annealing temperature. The probable reason is that the solubility of sodium bromide in the melt increases with temperature, and no solid precursors are in the samples after high-temperature calcination. At the same time, the relative intensity of the Cs_3_Bi_2_Br_9_ phase reflections (100) and (111) increases and becomes narrower with the sintering temperature according to the formation of larger crystallites of cesium bromobismuthates(III) when the melt is cooled from higher temperatures.

Also, a melt synthesis of the Cs_2_NaBiBr_6_ compound was carried out from a mixture of simple bromides at T = 650 °C using water quenching from a temperature of 450 °C. It was initially assumed that at T = 650 °C, the equilibrium state of the system is a liquid that provides a uniform distribution of elements in the sample. According to the diffraction data of such samples, the relative intensity of the sodium bromide reflections is significantly lower than that for the Cs_3_Bi_2_Br_9_ phase. This fact can be explained by the small size of the sodium bromide crystallites in the obtained sample in comparison with the sample obtained by standard cooling with a furnace. This result confirms the assumption that in the temperature range of 500–650 °C, sodium bromide crystals are predominantly dissolved in the melt; however, at T = 450 °C, the seeds of the Cs_3_Bi_2_Br_9_ compound are already present in the melt.

**Figure 2:**
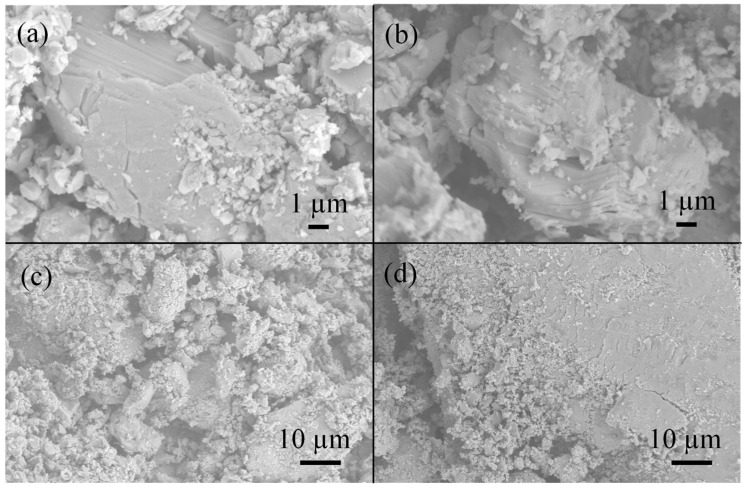
SEM micrographs of the samples of the theoretical composition Cs_2_NaBiBr_6_ obtained by ampoule synthesis at (**a**) T = 320 °C, (**b**,**c**) T = 500 °C, and (**d**) T = 650 °C.

A more detailed study of crystallization processes in the melt of bromides requires microscopic examination of samples. Figure 2 demonstrates the characteristic microstructure of the compositions obtained at 300 °C, 500 °C, and 650 °C with a standard cooling procedure. The micrographs show the presence of large single crystalline particles with rounded edges. The characteristic size of large single crystals grows with the temperature of exposition and is up to 100 µm when the temperature is 650 °C (see Figure 2a,d). The microstructure of individual grains is layered, which is evident on chipped crystals (Figure 2a,b). This most likely belongs to the (101) crystal planes which are the most intensive in the diffraction pattern for the Cs_3_Bi_2_Br_9_ phase.

**Figure 3:**
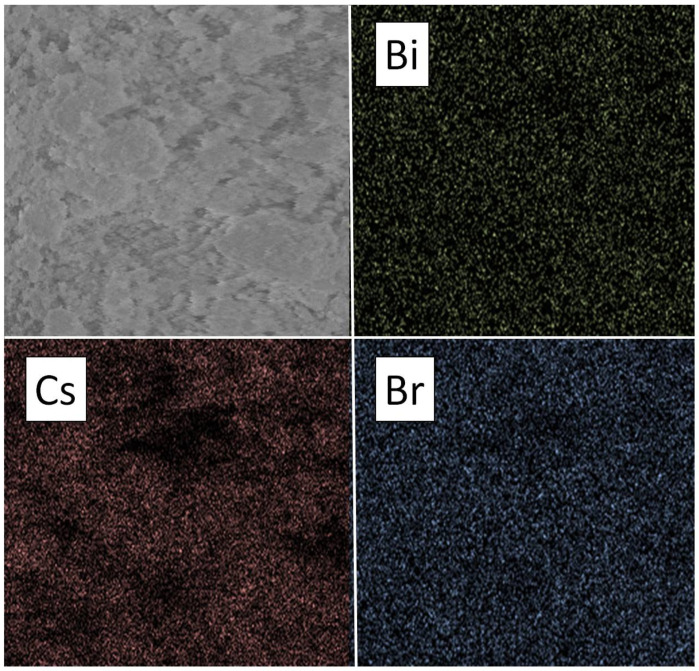
EDS mapping data (elements are Cs, Bi, Br) for the sample of the theoretical composition Cs_2_NaBiBr_6_ obtained by ampoule synthesis at T = 500°C.

The elemental analysis (EDS) of the powders of the theoretical composition Cs_2_NaBiBr_6_ confirmed the presence of all elements in samples with elemental ratios rather close to the expected ones. The elemental mapping (Figure 3) demonstrates an uneven distribution of bismuth and cesium, while the bromine distribution is uniform. The mapping images for Cs and Bi look rather similar, corresponding well to the XRD results.

**Figure 4:**
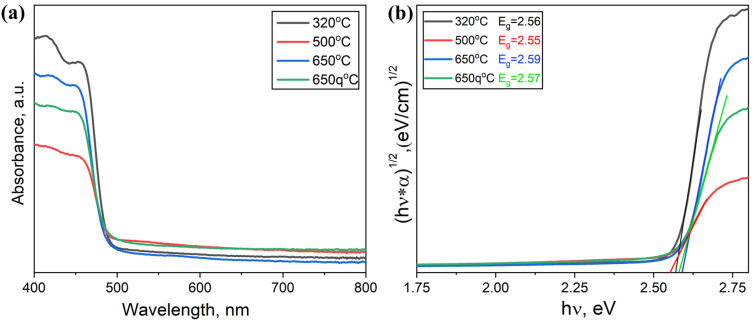
(**a**) Optical absorption spectra and (**b**) Tauc plots of optical density data with linear trends for samples of the theoretical composition Cs_2_NaBiBr_6_ obtained by heterophase synthesis at T = 320–650 °C with standard cooling and at 650 °C with quenching.

The optical properties of the samples are studied using diffuse reflectance spectroscopy and photoluminescence spectroscopy (Figure 4). The absorption spectra of the samples differ within the limits of the accuracy of the method and correspond to the spectra of non-single-phase samples. No evident dependence of optical spectra on temperature exists for the samples fused at 500 °C, 650 °C, and 650 °C with quenching. Containing the Cs_3_Bi_2_Br_9_ and NaBr phases, the samples produce two absorption edges in their DRS spectra. The Cs_3_Bi_2_Br_9_ phase makes the main contribution to the absorption edge region of the samples at about 475 nm (~2.6 eV). According to the literature [22,23,24], for the Cs_3_Bi_2_Br_9_ bromide, the bandgap is 2.5–2.7 eV. The absorption edge for the sodium bromide NaBr is at 6.12 eV [25]. The shoulder at 475 nm is the most intensive for the sample prepared by a solid-phase method, which makes melt crystallization a promising route for preparing uniform samples.

**Figure 5:**
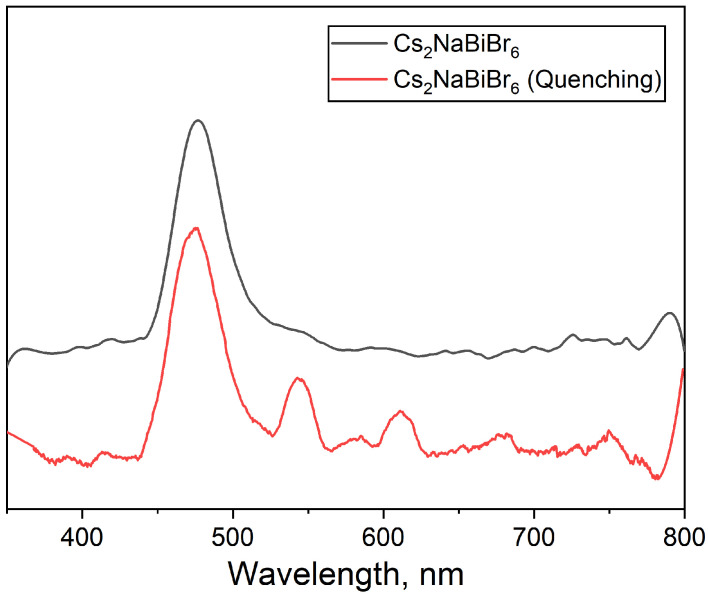
PL spectra of the samples with a general composition of Cs_2_NaBiBr_6_ obtained by heating at 650 °C with quenching or with a standard cooling process. T = 298 K, λ_ex_ = 337 nm.

Figure 5 shows a comparison of photoluminescence spectra of the samples of the theoretical composition Cs_2_NaBiBr_6_ obtained both by standard cooling from 650 °C to room temperature (black line) and using quenching (red line). The spectrum contains two clearly defined regions of PL, one at about 450–510 nm and another less intensive one at 510–530 nm, presumably consisting of two or more components. The first region can be attributed to the coarse crystalline Cs_3_Bi_2_Br_9_ phase [26,27]. According to the literature, the photoluminescence spectrum of this compound is represented by two maxima, one located at 477 nm and a weaker one at about 544 nm [28]. The following spectral maxima are too weak and broad to be interpreted correctly. The strongest maximum at 477 nm should be related to Cs_3_Bi_2_Br_9_ double bromide [28]. This also correlates with the absorption edge in optical spectra (see Figure 3) at about 475 nm. 

The PL spectrum of the quenched product includes four narrower and stronger peaks in the tail region. Namely, the most intense maximum at 475 nm and four less intense ones at about 540 nm, 580 nm, 610 nm, and 680 nm may indicate significant differences in the optical properties of the materials according to differences in their crystallinity. Presumably, the peak at 475 nm belongs to the near-band edge (NBE) emission for the Cs_3_Bi_2_Br_9_ phase and is similar to the 477 nm peak of the coarse crystalline Cs_3_Bi_2_Br_9_ phase. The weak maximum at 580 nm also belongs to the PL of Cs_3_Bi_2_Br_9_. Three PL maxima probably originated from point defects in Cs_3_Bi_2_Br_9_ [29,30] but also could be related to cesium and sodium bromides. An admixture of BiOBr could give its input at 450–480 nm.

The obtained results indicate that the melt crystallization process is far from the optimal conditions for obtaining a double perovskite Cs_2_NaBiBr_6_ since the binary bromide phase Cs_3_Bi_2_Br_9_ crystallizes in the temperature range T = 320–650 °C.

It can be assumed that the single-phase Cs_2_NaBiBr_6_ composition could be obtained by quenching the melt from higher temperatures (T = 600 °C or higher) or using soft chemistry methods at room temperature as described for some other complex halides with a double perovskite structure.

### 3.2. Samples of General Composition Cs_2_Ag_1−x_Na_x_BiBr_6_

**Figure 6:**
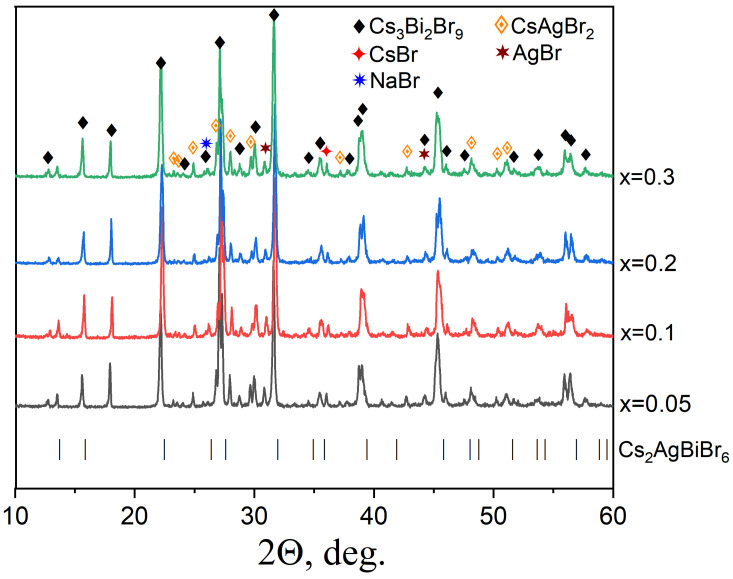
The XRD data for the samples of the general composition Cs_2_Ag_1−x_Na_x_BiBr_6_ (*x* = 0.05–0.3). Reflections of a double perovskite Cs_2_AgBiBr_6_ phase are given as lines.

Since the possibility of synthesizing the Cs_2_AgBiBr_6_ phase at low heat treatment temperatures is known from the literature, a solid-state synthesis at 300 °C was applied for the preparation of Na-substituted Cs_2_Ag_1−x_Na_x_BiBr_6_ double perovskites. All samples demonstrate the following strong characteristic reflections of Cs_2_AgBiBr_6_ double perovskite in XRD patterns in Figure 6: (200), (220), (222), (400), etc. We also see a shift of the characteristic reflection (022) at 2θ ~ 22.35° towards a smaller 2θ, as was observed by Li et al. [15] for the Sb-substituted Cs_2_AgBiBr_6_ phase.

Samples of the theoretical composition Cs_2_Ag_1−x_Na_x_BiBr_6_ with the degree of substitution *x* = 0.05, 0.1, 0.2, and 0.3 were obtained. According to the XRD data (Figure 5), the samples are not single-phase, contain Cs_2_AgBiBr_6_ and Cs_3_Bi_2_Br_9_ phases, and in smaller percentages, could present simple bromides AgBr (PDF-2 [83-1426]) and NaBr (PDF-2 [72-1539]), which are precursors. Many reflections overlap, which complicates a more detailed interpretation of the XRD data. It can be assumed that sodium bromide did not react completely and is present in the samples due to its low reactivity or was recrystallized from the melt. An admixture of bismuth (III) bromide may be present due to its volatility (T_m_ = 200–219 °C).

**Figure 7:**
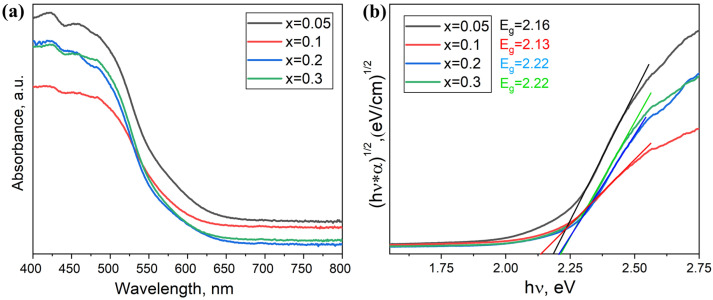
(**a**) Optical absorption spectra and (**b**) Tauc plots of optical density data with linear trends for samples of the general composition Cs_2_Ag_1−x_Na_x_BiBr_6_ (*x* = 0.05–0.3).

The estimated values of the optical bandgap for samples of the theoretical composition Cs_2_Ag_1−x_Na_x_BiBr_6_ (*x* = 0.05–0.3) are shown in Figure 7. The values of *Eg* are in the range of 2.16–2.22 eV, whereas for the Cs_2_AgBiBr_6_ phase, according to the literature, the bandgap is 2.07 eV. The calculated bandgap values are smaller for x = 0 and 0.1, while for the x = 0.2 and 0.3 compositions, it is 2.22 eV.

Since the observed spectra correspond well to the spectra of samples containing coarse crystalline Cs_3_Bi_2_Br_9_, the contribution to the optical spectrum from this phase is the most significant. According to the literature, its bandgap value is 2.56–2.62 eV [22,23,24]. It cannot be ruled out that the Cs_2_AgBiBr_6_ phase was nevertheless formed, but the absorption coefficient of the Cs_2_AgBiBr_6_ compound is significantly lower than that of the binary bromide Cs_3_Bi_2_Br_9_. In the presented spectra, we see the top of the absorption edge of the binary bromide Cs_3_Bi_2_Br_9_ at a minimum of about 450 nm.

**Figure 8:**
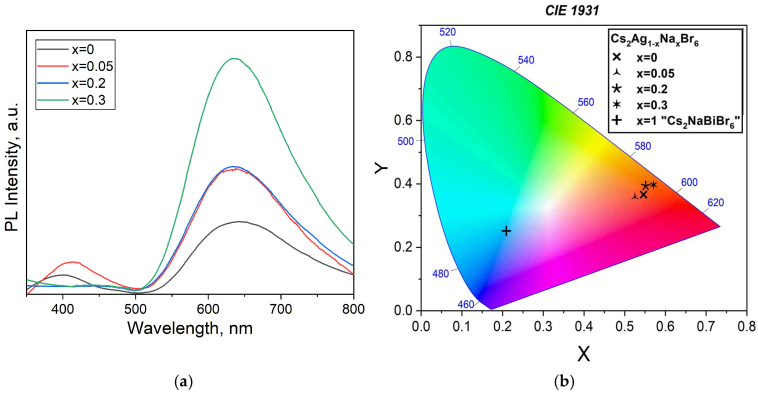
(**a**) Photoluminescence spectra of the samples of the theoretical composition Cs_2_Ag_1−x_Na_x_BiBr_6_ (*x* = 0–0.3). T = 298 K, λ_ex_ = 337 nm. (**b**) The chromaticity coordinates CIE 1931 of the samples of the theoretical composition Cs_2_Ag_1−x_Na_x_BiBr_6_ (*x* = 0–0.3). The coordinates of Cs_2_NaBiBr_6_ composition are given as *x* = 1.

Figure 8a shows the PL spectra of the Cs_2_Ag_1−x_Na_x_BiBr_6_ samples at different substitution rates, namely *x* = 0, *x* = 0.05, *x* = 0.2, and *x* = 0.3. An increase in the substitution rate *x* produces the shifting of the absorption peak of maximum intensity toward shorter wavelengths (so-called “blue shift”), and the peak becomes broader. This may indicate a change in the electronic structure as a result of deficiency growth.

For the Cs_2_AgBiBr_6_ phase sample (*x* = 0), the intensive peak is located at about 650 nm, which corresponds to the literature. For the Na-substituted composition *x* = 0.05, the intensity peak shifts slightly to the left and has its maximum at 640 nm. For the *x* = 0.2 composition, the peak shifts even further to the blue region (to about 630 nm) with a further increase in intensity. For the *x* = 0.3 composition, the intensity peak is located still at about 630 nm but has the highest photoluminescence intensity of the entire group of samples. It can be assumed that the shift in the maximum position is a consequence of the formation of a substitution solid solution with the double perovskite Cs_2_AgBiBr_6_ structure. This supposition could be confirmed by the results of calculating the unit cell parameter of double perovskite for these samples.

The luminance–chromaticity color space for the samples of the theoretical composition Cs_2_Ag_1−x_Na_x_BiBr_6_ (*x* = 0–0.3) is shown in Figure 8b. The diagram makes it evident that the photoluminescence of the samples Cs_2_Ag_1−x_Na_x_BiBr_6_ changes step by step with sodium content. Regarding the phase composition of Cs_2_Ag_1−x_Na_x_BiBr_6_, we believe that the formation of substitution solid solutions could take place.

As a result, we observed a correlation between the optical properties of the Cs_2_Ag_1−x_Na_x_BiBr_6_ (*x* = 0–0.3) compounds and their chemical compositions. This leads to optimism about the presence of a double perovskite phase in the samples and the opportunity to change its optical characteristics. The collected PL spectra of the samples most likely correspond to Cs_2_Ag_1−x_Na_x_BiBr_6_ solid solutions. A number of simple or double bromides could also be present in the samples but contribute to their optical and PL spectra as admixtures only.

### 3.3. Samples of General Composition Cs_2−2x_Na_x_AgBiBr_6_

**Figure 9:**
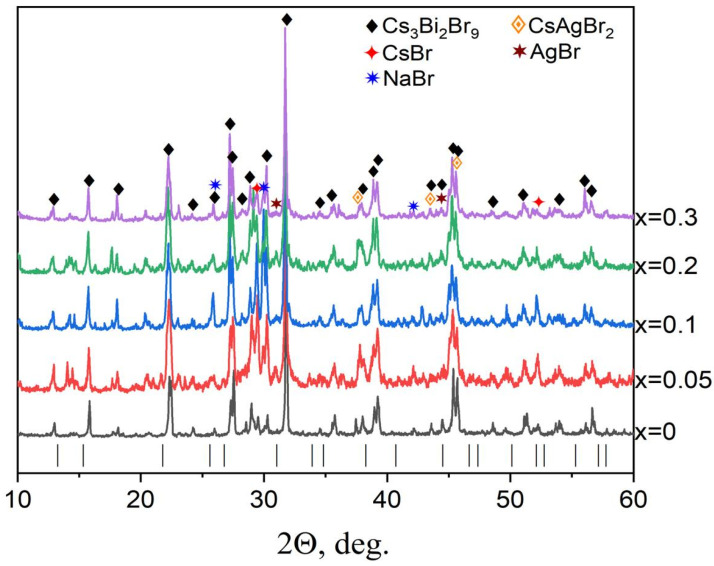
XRD data for the samples of the general composition Cs_2−2x_Na_x_AgBiBr_6_ (*x* = 0–0.3). Reflections of a double perovskite Cs_2_AgBiBr_6_ phase are shown as lines.

To analyze the influence of the cesium percentage on melt crystallization products, we analyzed the third group of samples with a varied cesium percentage. Samples of the theoretical composition Cs_2−2x_Na_x_AgBiBr_6_ (*x* = 0–0.3) were obtained by the melt method at annealing temperature T = 650 °C with reaction cooling, similar to those described in the literature [31]. The XRD method showed that the samples have a mixed composition, including the phases Cs_2_AgBiBr_6_, Cs_3_Bi_2_Br_9_, Cs_3_BiBr_6_ [32], and NaBr (Figure 9). It is worth noting that there is conflicting information in the literature regarding the supposed phase Cs_3_BiBr_6_. In some sources, the phase is indicated as equilibrium and stable [33], while in others, its instability is indicated compared to the phase Cs_3_Bi_2_Br_9_ [34].

**Figure 10:**
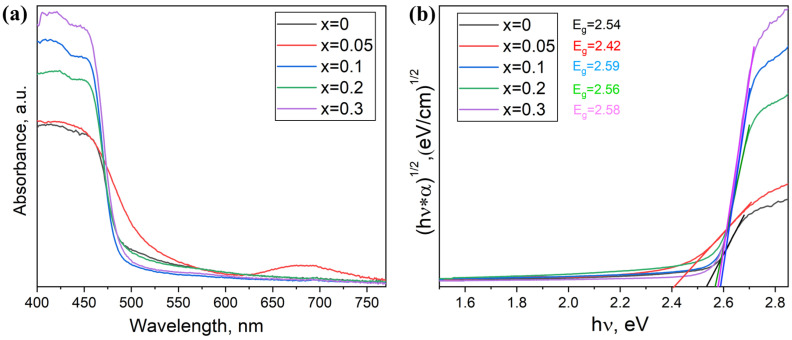
(**a**) Optical absorption spectra and (**b**) Tauc plots of optical density data for samples of the general composition Cs_2−2x_Na_x_AgBiBr_6_ with *x* = 0–0.3.

The optical spectra of the samples are shown in Figure 10. The estimated values of the optical bandgap for the samples are 2.54–2.59 eV, which is close to the bandgap value for double bromide Cs_3_Bi_2_Br_9_. Contradicting the Cs_2_Ag_1−x_Na_x_BiBr_6_ data, the absorption by the double perovskite phase Cs_2_AgBiBr_6_ is observed in the tail region of the spectra in the wavelength range of 500–600 nm. This also correlates with our XRD results.

Summarizing the results of melt crystallization of the samples of the general composition Cs_2−2x_Na_x_AgBiBr_6_ (*x* = 0–0.3), we can conclude that there is an increase in the cesium percentage shift.

## 4. Conclusions

In summary, it was demonstrated that the classic melt synthesis of perovskite bromide Cs_2_NaBiBr_6_ from simple bromides leads to the crystallization of Cs_3_Bi_2_Br_9_ (primary) and NaBr phases. The growth of micron-size (101)-textured Cs_3_Bi_2_Br_9_ crystals decreases the cesium percentage in the melt and prevents the precipitation of the Cs_2_NaBiBr_6_ phase in the temperature range of 400–650 °C. At the same time, it is worth noting that annealing at 650 °C leads to the complete dissolution of sodium bromide in the melt. Presumably, the double perovskite phase Cs_2_NaBiBr_6_ could have a decomposition temperature below 300 °C, and the phase could be synthesized successfully by fusing the ampoules at lower temperatures.

The synthesis of samples of a double perovskite phase Cs_2_AgBiBr_6_ and substituted compositions Cs_2_Ag_1−x_Na_x_BiBr_6_ resulted in non-single-phase products also containing the binary bromides Cs_3_Bi_2_Br_9_ and CsAgBr_2_ and impurities of the precursors AgBr and NaBr. This happens because of a lack of cesium in the melt, in the same way as observed for the melt of Cs_2_NaBiBr_6_. The optical bandgap values of the Cs_2_Ag_1−x_Na_x_BiBr_6_ samples are generally close to the literature values for the Cs_2_AgBiBr_6_ phase. It is shown that the position of the photoluminescence maximum of the Cs_2_AgBiBr_6_ phase shifts to the blue region for compositions with *x* = 0–0.2 with an increase in the sodium bromide content. This may indicate the substitution solid solution formation within 20%. The synthesis of samples of the theoretical composition Cs_2−x_Na_x_AgBiBr_6_ showed a formation of mixed compositions, presumably containing the Cs_2_AgBiBr_6_ phase, as well as impurities of Cs_3_Bi_2_Br_9_, Cs_3_BiBr_6_, and NaBr. So, the substitution of Cs by Na described in the literature for the double perovskite phase Cs_2_AgBiBr_6_ was not observed.

## Data Availability

The original contributions presented in this study are included in the article. Further inquiries can be directed to the corresponding author.
